# Cerebellar tDCS combined with augmented reality treadmill for freezing of gait in Parkinson’s disease: a randomized controlled trial

**DOI:** 10.1186/s12984-024-01457-z

**Published:** 2024-09-28

**Authors:** Fabrizio Pisano, Denise Mellace, Ambra Fugatti, Edoardo Nicolò Aiello, Silvia Diotti, Beatrice Curti, Alessandra Giust, Angelica Marfoli, Cecilia Perin, Angelica De Sandi, Dario Alimonti, Alberto Priori, Roberta Ferrucci

**Affiliations:** 1 Neurological Rehabilitiation, Policlinico San Marco, Gruppo San Donato, Zingonia, Bergamo Italy; 2 Department of Oncology and Hemato-Oncology, University of Milan, Via Santa Sofia 9/1, Milan, 20122 Italy; 3 Department of Neurology and Laboratory of Neuroscience, IRCCS Istituto Auxologico Italiano, Milan, Italy; 4 School of Medicine and Surgery, University of Milan-Bicocca, Milan, Italy; 5 Foundation IRCCS Ca’ Granda Ospedale Maggiore Policlinico, Milan, Italy; 6 Neurology Unit, Department of Neuroscience, ASST Papa Giovanni XXIII Hospital, Bergamo, Italy; 7 Neurological Clinic, ASST-Santi Paolo e Carlo, Department of Health Sciences, University of Milan, Milan, Italy; 8 “Aldo Ravelli” Center for Neurotechnology and Brain Therapeutics, Department of Health Sciences, University of Milan, Milan, Italy

**Keywords:** Freezing of gait, Cerebellum, Transcranial direct current stimulation, Physical exercise, Augmented reality, Rehabilitation

## Abstract

**Background:**

Parkinson’s disease (PD) is often accompanied by gait disorders and freezing of gait (FoG), disabling symptoms that are resistant to conventional dopamine treatments. Given the cerebellum’s connectivity with the motor cortex and basal ganglia, and its implication in PD, combining transcranial direct current stimulation targeting the cerebellum (ctDCS) with physical exercise might improve gait and balance.

**Objective:**

This study aimed to evaluate the effectiveness of a novel rehabilitation approach that combines noninvasive cerebellar stimulation with motor-cognitive training via an augmented reality treadmill (C-Mill VR^+^) in individuals with PD and FoG.

**Methods:**

Seventeen individuals with PD exhibiting FoG were enrolled in a randomized controlled trial. The participants were randomly assigned to a group receiving motor-cognitive training on the C-Mill VR^+^ with either ctDCS or sham ctDCS. Assessments were conducted pre-intervention (T0), post-intervention (T1) after 10 sessions, and at 4-week follow-up (T2), using various clinical scales. Additionally, C-Mill assessments of postural stability and gait were conducted at T0 and T1.

**Results:**

Although no significant time*group interactions were observed for any of the clinical variables measured, some were found in the C-Mill measures. Specifically, right lower limb sway in static conditions, both with eyes open (OAD) and eyes closed (OCD), significantly improved at T1 in the ctDCS group compared with the sham group.

**Conclusions:**

C-Mill outcomes indicate that the combined treatment may enhance motor control. Participants who received ctDCS along with augmented reality motor-cognitive training showed better postural stability.

**Supplementary Information:**

The online version contains supplementary material available at 10.1186/s12984-024-01457-z.

## Introduction

Parkinson’s disease (PD) is the second most common progressive neurodegenerative condition [1], and results from the degeneration of dopaminergic neurons in the substantia nigra, leading to dopamine deficiency [2]. It is characterized by both motor and nonmotor symptoms. Common motor symptoms include resting tremor, rigidity, and bradykinesia, and as the disease progresses, postural instability and gait disorders occur [3]. Freezing of gait (FoG), a prominent manifestation of impaired walking in people with PD, contributes to debilitating falls and significantly impacts quality of life [4]. The prevalence of FoG in people with PD can reach 63%, with its occurrence typically escalating in the advanced stages of the disease [5]. FoG is described as a brief episode of inability to walk smoothly, often triggered during activities such as turning, passing in narrow spaces, dual-tasking, and in stressful situations [6]. Although structural and functional abnormalities in cortical and subcortical brain regions have been linked to FoG pathogenesis [7, 8], there is limited consensus on the singular anatomical region responsible for it. Recent network-based analyses suggest that FoG in PD arises from a complex disorder involving multiple brain networks [5, 9]. In addition to the well-known dysfunction of the basal ganglia and its connections with cortical pathways, the cerebellum also plays a significant role in the development of both motor and nonmotor symptoms in PD [9]. Currently, there is no effective pharmacological treatment for FoG, and deep brain stimulation (DBS) has shown unsatisfactory results in relieving FoG symptoms. As a result, research has increasingly focused on nonpharmacological and nonsurgical therapies to improve the quality of life of individuals with PD [10]. Exercise and physical training have demonstrated positive effects on mobility, balance, affective status, and quality of life [3]. Among these interventions, treadmill training, such as aerobic exercise on an augmented reality treadmill (C-Mill VR^+^, Hocoma Motek), has shown positive effects on gait and freezing [3, 11, 12]. The C-Mill is an advanced treadmill for gait and balance assessment and training, that integrates augmented virtual reality, audible and visual cues, and force platform technology, enabling obstacle avoidance training, dual-tasking exercises, and immersive virtual reality environments and promoting balance strategies and gait adaptation in a safe setting [13].

Among neuromodulation interventions, transcranial direct current stimulation (tDCS) is a noninvasive brain stimulation technique that can modify cortical excitability [14] and has been shown to foster motor skill acquisition [15] and yield lasting beneficial effects on motor performance [10]. Modulating dysfunctional brain regions associated with FoG through tDCS could alleviate symptoms by normalizing neural activity [16, 17]. Advances in technology, including wearable sensors, virtual reality, and portable stimulation devices such as tDCS, have facilitated the development of more effective therapeutic options [4]. Research indicates that combining neuromodulation with treadmill training is more effective in treating gait dysfunction, particularly in reducing FoG, than is conventional training alone [4, 16]. Targeting the cerebellum with tDCS—given its connection with the motor cortex and basal ganglia, and its involvement in PD [18]—combined with physical exercise holds promise for improving gait and balance [19].

The primary aim of this study was to evaluate whether the combination of the motor-cognitive training via C-Mill VR^+^ and cerebellar tDCS (ctDCS) can provide additional improvements in motor, functional and cognitive function in individuals with PD, specifically by exploring their combined effects on FoG.

## Methods

### Study design

This study is a single-blind, prospective, single-center, randomized clinical trial (RCT), conducted in the Neurorehabilitation Unit of Policlinico San Marco in Osio Sotto, Bergamo, Italy, a specialized center for assisting to individuals with PD, beginning in May 2023. The study was carried out according to the principles of the Declaration of Helsinki and good clinical practice standards and in line with the Standard Protocol Items Recommendations for Interventional Trials (SPIRITS) guidelines. The Ethics Committee of the ASST Papa Giovanni XXIII Hospital, Bergamo, approved all the experimental procedures on 11th May 2023. All the individuals who agreed to participate provided written informed consent.

### Participants

A group of 20 individuals with PD afferent to the outpatient clinic of Neurology, the VAMP Center, and the O.U. of Neurological Rehabilitation of Policlinico San Marco was enrolled.

Individuals were included if they had: idiopathic PD diagnosis according to the UK PD Society Brain Bank criteria, presence of FoG based on the neurologist’s clinical observation, disease staging ≥ 2 points according to the Hoehn & Yahr stage [20], age between 40 and 85 years, not exhibiting other associated neurological diseases and/or musculoskeletal and cardiorespiratory conditions, and no relevant cognitive deficits on the Montreal Cognitive Assessment (MoCA) [21] test. The exclusion criteria included a diagnosis of atypical PD, neuropsychiatric comorbidities, age younger than 18 years, not having undergone treatment rehabilitation in the previous three months, prior medical history of epilepsy and traumatic brain injury, neurosurgery, and the presence of pacemakers and DBS.

### Experimental procedure

The enrolled individuals with PD exhibiting FoG underwent gait training on the C-Mill VR^+^ associated with or not associated with ctDCS stimulation. The treatment consisted of one 20-minute session per day for 10 days, from Monday to Friday each week. All assessments were conducted by physicians and neuropsychologists with expertise in the management of PD and cognitive and motor assessments.

The participants were randomly allocated to either a group receiving motor-cognitive training on the C-Mill paired with ctDCS or with sham tDCS and were assessed at 3 timepoints: pre-intervention (T0), post-intervention (T1), and 4 weeks after the last intervention session (T2). Randomization for assignment to the group was performed via an online generator (https://www.random.org/lists/*)*, with odd and even numbers identifying subjects undergoing ctDCS and sham-tDCS treatment, respectively. The participants and caregivers were not aware of group allocation throughout the study. Physical therapists were the only ones aware of the type of treatments, as they must set the stimulation based on the randomization list. Notwithstanding, the treatment executor was instructed not to reveal the group assignment to anyone.

### Anodal ctDCS and motor-cognitive training

The participants underwent a comprehensive treatment program consisting of one 20-minute session per day for 10 days. These sessions involved the application of tDCS combined with a gait rehabilitation program. During the sessions, a portable battery-powered neurostimulator (BrainStim, E.M.S srl) delivered a direct current of 2 mA through two electrodes positioned at the cerebellar level, on the posterior cranial fossa (anode), and at the right arm (cathode). The neurostimulator was placed in a backpack to allow movement during treadmill training. The electrodes, each measuring 35 cm^2^, were arranged in a unipolar montage and covered with saline-soaked sponges and electrogel to optimize conductivity [22]. This ctDCS protocol has been used in other studies previously [22, 23].

For those in the sham group, the stimulation program consisted of 2 s of initial stimulation followed by a decrease until its shutdown, which was intended to mimic the initial sensations of real stimulation but without delivering continuous stimulation. This approach was adopted to provide a similar sensory experience to the participants while not providing therapeutic current.

The safety of the ctDCS application was a priority and was therefore assessed at each session by collecting information on perceived sensations, possible discomfort, or side effects.

In association with tDCS, each participant underwent a structured gait rehabilitation program via the C-Mill, which involved one 20-minute sessions per day, structured as follows: 10 min of gait training and 10 min of motor-cognitive training. During gait training on the C-Mill, participants engaged in various exercises designed to improve their walking pattern and adaptability. This included the following C-Mill exercises: (1) gait assessment (2 min); (2) gait adaptability (3 min); (3) stepping stones random (3 min), in which the subject had to reach and step rectangular-shaped targets projected on the treadmill, which could transform into an obstacle, necessitating adjustment in their walking pattern to avoid it; and (4) speed adaptability (2 min), in which the subject has to walk inside a green rectangle that moves back and forth on the carpet, forcing the subject to speed up and slow down to stay inside the rectangular perimeter.

The motor-cognitive training component of the program focused on enhancing participants’ ability to perform dual tasks while walking. This included the following C-Mill exercises: (1) Trace (3 min): subjects had to interact with objects appearing on the treadmill while walking, by stepping onto them or avoiding them; (2) Soccer walking (3 min): while walking, subjects had to control a virtual ball using lateral movements, without dropping the ball to the ground; and (3) Italian Alps (4 min): while walking, subjects collected ingredients to make pizza by moving left and right.

### Outcome measures

Assessments were performed on day 1 (T0), before the intervention began; on day 10 (T1), immediately after the last intervention session; and four weeks after the completion of the treatment (T2). All assessments were performed in the ON state of PD medication.

Participants were assessed at T0, T1 and T2 via the following scales/instruments: part 3 of the Unified Parkinson’s Disease Rating Scale (UPDRS-III) [20] to evaluate general motor functions; the Freezing of Gait Questionnaire (FOG-Q) [24]; the 6 min Walk Test (6MWT) [25] to assess endurance; the Borg Category Ratio Scale 0–10 (BORG) [26] to assess the perception of exertion; the Mini Balance Evaluation Systems Test (Mini-BESTest) [27] Italian version, to detect balance impairments; the Timed Up and Go (TUG) test [28] to assess functional mobility, balance, walking, and fall risk; the MoCA test [21] and Mini Mental State Examination (MMSE) [29] to evaluate overall cognitive performance; the Frontal Assessment Battery (FAB) [30]; the Parkinson’s Disease Questionnaire-8 (PDQ-8) [31] to assess quality of life; the Beck Depression Inventory-II (BDI-II) [32] Italian version to detect the severity of depressive symptoms; and the Barthel Index (BI) [33], the Activities of Daily Living (ADL) [34] and Instrumental Activities of Daily Living (IADL) [35] to monitor functional changes and autonomy in daily life.

Motor function was also assessed by C-Mill assessments at T0 and T1, which allow the evaluation of postural control and gait through the recording of the following parameters:

Stand assessment for postural control:

- Limits of Stability, which measures how far a subject can lean safely in different directions without losing balance as an indicator of dynamic stability. The recorded indicators include the surface area within which the Center of Pressure moves as a person tries to maintain balance (Sup CoP), as well as its oscillations along the medio-lateral (ML) and antero-posterior (AP) axis.
- Postural Stability, which evaluates body sway (CoP velocity) in various static positions as an indicator of balance and postural control while standing: body sway on the right while standing with eyes open (OAD) and on the left (OAS), body sway on the right lower limb while standing with eyes closed (OCD) and on the left (OCS), body sway on the right in tandem stance (TD) and on the left (TS), and single-leg stance to the right leg (Dx) and to the left leg (Sin).

Gait assessment:

- Gait Assessment, which measures the subject’s walking pattern on a treadmill during a 5-minute walking, during which the C-Mill records parameters such as right step length (LPDx), left step length (LPSx), step width (AmP), right weight distribution (DPDx), and left weight distribution (DPSx).
- Gait Adaptability, which assesses the ability to navigate obstacles on the treadmill. The recorded parameters include right step length with obstacles (LPDxO), left step length with obstacles (LPSxO), step width with obstacles (AmPO), right weight distribution with obstacles (DPODx), and left weight distribution with obstacles (DPOSx).

### Statistics

#### Cognitive and motor-functional outcomes

The majority of the cognitive and motor-functional outcomes across the three time-points, proved to distribute Normally (i.e., skewness and kurtosis values >|1| and >|3|, respectively [36]), except for the BI, TUG, ADL and MMSE scores, which were effectively normalized *via* a reverse transformation. Hence, the effects of *Time*, *Group* and their interaction (Time*Group) on each cognitive and motor-functional outcome were explored *via* either linear or generalized linear mixed models by assuming different underlying data-generating processes on the basis of empirical data distributions. More specifically, the UPDRS-III, FOG-Q, MoCA, FAB, PDQ-8, BDI, BORG, Mini-BEST, 6MWT and IADL scores were analyzed *via* linear mixed models (i.e., assuming an underlying Normal distribution), whereas the BI, TUG, ADL scores were analyzed *via* generalized linear mixed models underlying Negative Binomial distributions, and the MMSE score was analyzed *via a* generalized linear mixed model underlying a Gamma distribution – which is a data-generating process suitable for empirical distributions characterized by floor-like effects and high interindividual variability [37]. Zero values are not allowed to fit a Gamma distribution, so a constant *K* = 0.01 was added to the MMSE score. Within all of these models, *Subject* was addressed as the cluster, whereas *Time* and *Group* were addressed as between- and within-subject factors, respectively. A random intercept was fitted within the *Subject* cluster. Bonferroni-corrected post-hoc comparisons were run for significant terms. We focused only on interaction effects.

#### C-Mill outcomes

The vast majority of C-Mill measures across the two time-points were distributed Normally, as yielded by the abovementioned descriptive analysis, with the exception of OAS, OCS, TD, TS, Dx, DPDx, and MLr, which were effectively normalized *via* a reverse transformation. Hence, the effects of *Time*, *Group* and their interaction (Time*Group) on each C-Mill outcome were explored *via* either linear or generalized linear mixed models. Different underlying data-generating processes were assumed based on empirical data distributions. More specifically, SupCoP, AP, OAD, OCD, Sin, LPDx, LPSx, AmP, DPSx, LPDxO, LPSxO, AmPO, DPODx, and DPOSx were analyzed *via* linear mixed models (i.e., assuming an underlying Normal distribution), whereas OAS, OCS, TD, TS, Dx, DPDx, and MLr, all being moderately to heavily right-skewed and overdispersed, were analyzed *via* generalized linear mixed models underlying a Gamma distribution, which is a data-generating process suitable for empirical distributions characterized by floor-like effects and high interindividual variability [37]. Zero values are not allowed to fit a Gamma distribution, so a constant *K* = 0.01 was added to the above variables. Within all of these models, *Subject* was addressed as the cluster, whereas *Time* and *Group* as between- and within-subject factors, respectively. A random intercept was fitted within the *Subject* cluster. Bonferroni-corrected post-hoc comparisons were run for significant terms. We focused only on interaction effects.

Analyses were run *via* IBM^®^ SPSS^®^ Statistics 29 (IBM Corp., 2023) and jamovi 2.3 (the jamovi project, 2022). Missing data were excluded pairwise in accordance with a per-protocol analysis.

## Results

### Background analyses

**Fig. 1:**
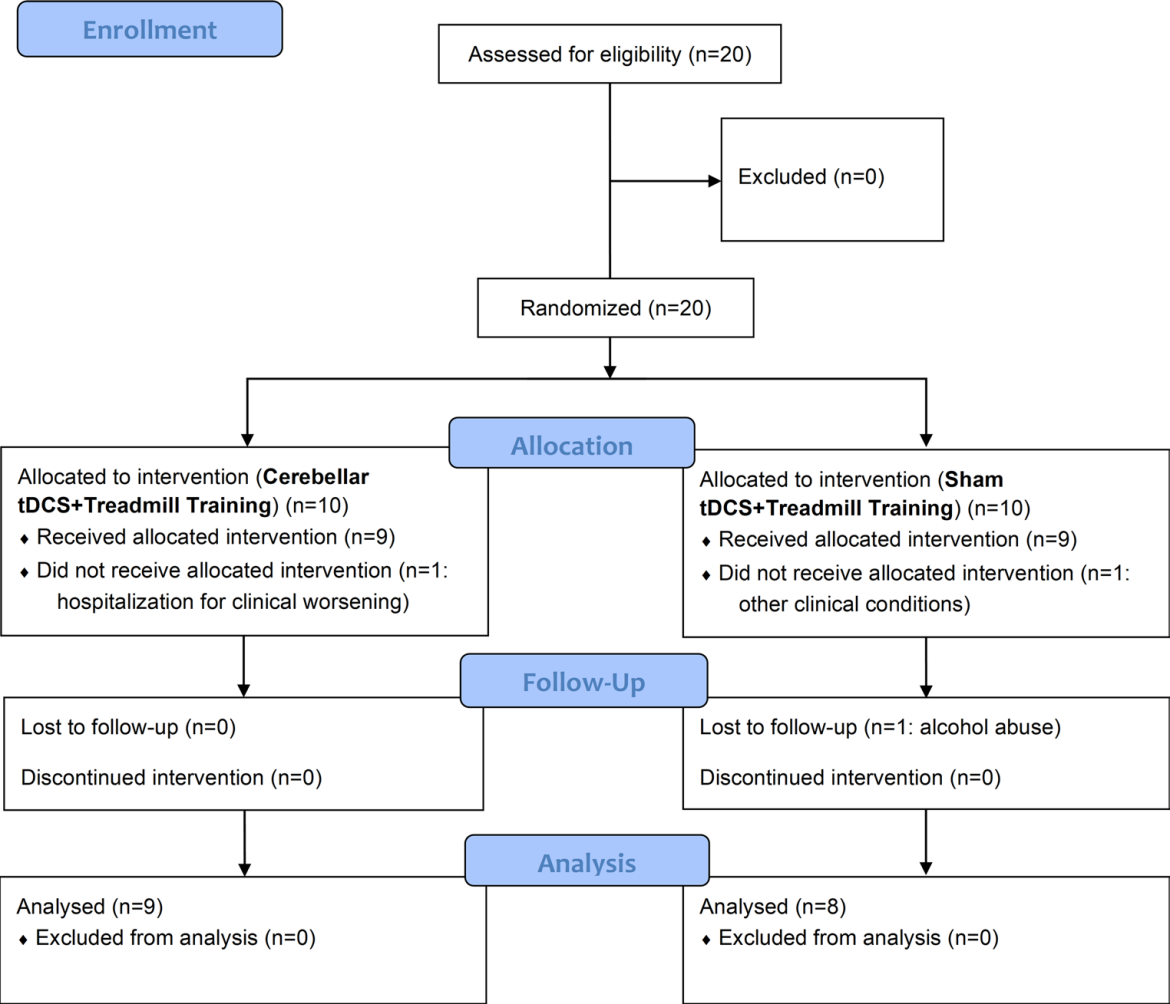
Flow diagram of participants in the study

Seventeen individuals with PD completed the study (Fig. 1). One of the initially selected subjects was hospitalized due to clinical worsening, a second subject was excluded at follow-up because he had begun abusing alcohol, and a third was injured due to a fall and could not complete the rehabilitation process. The ctDCS group included 9 subjects (3 males and 6 females), and the sham-tDCS group included 8 subjects (4 males and 4 females).

Table 1 summarizes the participants’ demographic data. The two groups were matched for age, sex, education and H&Y scale score. All participants were assessed in the ON state of PD medication.

**Table 1:** Participants’ background and clinical measures at baseline

	Cerebellar Group	Sham Group	*p*
*N*	9	8	
Age (years, Mean ± SD)	71 ± 8.6 (57–82)	65.3 ± 8.5 (48–75)	.210^a^
Sex (male/female, %)	18%/35%	24%/24%	.486^b^
Education (years, Mean ± SD)	9.3 ± 3.8 (5–12)	10.6 ± 4.1 (8–17)	.646^a^
H&Y (2/2.5/3, %)	24%/12%/18%	24%/12%/12%	.932^b^

*Notes *^a^Mann-Whitney’s *U*-statistic; ^b^χ^2^-statistic; H&Y = Hoehn and Yahr scale

### Cognitive and motor-functional outcomes

Table 2 shows the participants’ psychometric scores across the three time-points, whereas Supplemental Table 1 reports the results of the repeated-measures models. No measure had at-random missing data except for the TUG (one subject receiving cerebellar ctDCS). No significant interaction effects were found.

**Table 2:** Participants’ cognitive and motor-functional scores across the three time-points

	tDCS groups	T0 (Mean ± SD)	T1 (Mean ± SD)	T2 (Mean ± SD)
UPDRS-III				
	Cerebellar	42.1 ± 14.1 (18–67)	34.7 ± 12.2 (10–45)	34.2 ± 13.4 (5–49)
	Sham	31.5 ± 10.7 (16–47)	25.4 ± 8.6 (13–37)	26.8 ± 11.5 (8–43)
FoG-Q				
	Cerebellar	12.1 ± 3.2 (9–18)	8.3 ± 2.1 (5–12)	7.7 ± 2.9 (3–12)
	Sham	11.5 ± 3.3 (7–16)	6.8 ± 3.3 (2–11)	8.3 ± 4.3 (2–15)
MoCA				
	Cerebellar	23 ± 5.1 (15.1–30)	23.5 ± 3.1 (20.1–28.4)	23.5 ± 4.1 (17.6–29.4)
	Sham	22.8 ± 3.6 (14.7–26.5)	23.3 ± 4 (15.7–28.3)	23 ± 4.3 (16.7–30)
MMSE				
	Cerebellar	27.3 ± 3.3 (21.5–30)	27.3 ± 2.5 (21.5–30)	27.2 ± 2.8 (21.2–30)
	Sham	27.1 ± 3 (20.2–30)	27.5 ± 2.3 (23.2–30)	27.2 ± 2.9 (21.2–30)
FAB				
	Cerebellar	14.6 ± 2.6 (9.6–18)	14.9 ± 3 (10.6–18)	15.8 ± 2.4 (11.6–18)
	Sham	14.9 ± 3.9 (7.4–18)	15.5 ± 2.9 (9.5–17.9)	15.5 ± 2.5 (11.5–18)
PDQ-8				
	Cerebellar	8.9 ± 5.9 (1–21)	9.1 ± 5.7 (3–20)	9.2 ± 6.6 (2–23)
	Sham	7.6 ± 5.8 (2–19)	6.3 ± 6.5 (1–19)	7.8 ± 6.7 (2–18)
BDI				
	Cerebellar	12.3 ± 4.9 (5–21)	10.2 ± 5.6 (1–20)	11.3 ± 7.3 (0–21)
	Sham	8.9 ± 4.5 (4–15)	8.5 ± 7 (2–23)	8.6 ± 7.5 (1–21)
BORG_pre				
	Cerebellar	10.3 ± 4 (5–18)	8.7 ± 3.4 (5–16)	9.7 ± 3.8 (5–16)
	Sham	8.4 ± 3.7 (6–15)	8.1 ± 2.6 (6–13)	7 ± 1.9 (6–11)
BORG_post				
	Cerebellar	13.8 ± 3.8 (6–20)	12.9 ± 3.6 (6–19)	13.6 ± 3.1 (7–19)
	Sham	11.3 ± 3.3 (7–17)	11.8 ± 2.1 (8–15)	10.9 ± 1.8 (8–13)
Mini-BEST				
	Cerebellar	15.7 ± 6.2 (5–23)	18.2 ± 5.7 (7–25)	18 ± 6.4 (5–25)
	Sham	17.6 ± 5.3 (10–24)	21.6 ± 3.2 (17–26)	21.6 ± 5.1 (14–28)
6MWT				
	Cerebellar	262.5 ± 81.5 (143.4–400)	321.6 ± 96.2 (198.5–460)	300.2 ± 121.5 (115.2–460)
	Sham	374.5 ± 48.5 (325–480)	392.9 ± 53 (327–496)	400.3 ± 34.6 (354-462.5)
TUG				
	Cerebellar	12.2 ± 3.6 (8.1–18)	11.7 ± 4.3 (6.6–21.2)	14.2 ± 7.8 (7.9–31)
	Sham	11.4 ± 1.6 (7.8–15)	9.4 ± 1.9 (7.1–12)	10.2 ± 2.3 (7.6–13)
BI				
	Cerebellar	72.7 ± 16.9 (34–94)	77.8 ± 11.8 (63–94)	76.6 ± 12.4 (64–94)
	Sham	89.6 ± 9.2 (71–98)	94.4 ± 3.7 (87–100)	93.9 ± 4.5 (85–100)
ADL				
	Cerebellar	3.8 ± 1.4 (1–5)	4.1 ± 1.5 (1–6)	3.9 ± 1.5 (1–5)
	Sham	5.5 ± 0.8 (4–6)	5.8 ± 0.5 (5–6)	5.8 ± 0.5 (5–6)
IADL				
	Cerebellar	4 ± 2.3 (1–8)	3.8 ± 2.4 (1–8)	3.7 ± 2.5 (1–8)
	Sham	4.9 ± 2 (2–8)	4.9 ± 2 (2–8)	4.9 ± 2 (2–8)

*Notes* ADL = Activity of Daily Living; BDI = Beck Depression Inventory; BI = Barthel index; FAB = Frontal Assessment Battery; FoG-Q = Freezing of Gait Questionnaire; IADL = Instrumental Activity of Daily Living; MoCA = Montreal Cognitive Assessment; MMSE = Mini Mental State Examination; Mini-BEST = Mini Balance Evaluation System Test; 6MWT = 6 min Walking Test; PDQ-8 = Parkinson’s Disease Questionnaire; TUG = Time Up and Go Test; UPDRS-III = Unified Parkinson’s Disease Rating Scale–Part III

### C-Mill outcomes

Table 3 summarizes participants’ C-Mill scores across the two time-points, whereas Supplemental Table 2 reports the results of mixed models addressing such outcomes. At-random missing data were present solely for two subjects (one receiving sham stimulation and the other ctDCS) for the following measures: LPDxO, LPSxO, AmPO, DPODx and DPOSx.

**Table 3:** Participants’ C-Mill scores across the two time-points

	tDCS groups	T0 (Mean ± SD)	T1 (Mean ± SD)
SupCoP			
	Cerebellar	117.8 ± 51.4 (56.2-201-4)	161.2 ± 83.8 (0-259.5)
	Sham	144.8 ± 73.6 (24.7-276.2)	163.7 ± 65.5 (67.2-264.8)
ML			
	Cerebellar	19.5 ± 3.9 (13.1–23.5)	20.1 ± 8.3 (0-28.8)
	Sham	22.3 ± 4.1 (15.6–28.8)	23.7 ± 3.6 (19.6–28.8)
AP			
	Cerebellar	11.8 ± 3.8 (7.4–17.7)	14.9 ± 8.6 (1.8–30.4)
	Sham	15.2 ± 4.1 (9.6–22.4)	13.9 ± 5 (6.6–18.9)
OAD			
	Cerebellar	1.29 ± 2 (0-4.8)	5 ± 3.2 (0-9.7)
	Sham	2.7 ± 2.1 (0-6.3)	1.2 ± 1.7 (0-3.6)
OAS			
	Cerebellar	3.9 ± 1.7 (2.4-8)	4.5 ± 2.6 (2-10.5)
	Sham	3.4 ± 0.8 (2.6–5.1)	3.4 ± 0.6 (2.7–4.4)
OCD			
	Cerebellar	1 ± 2.2 (0-6.1)	4 ± 2.7 (0-9.9)
	Sham	2.5 ± 1.7 (0-4.3)	1.4 ± 2.1 (0-5.1)
OCS			
	Cerebellar	5 ± 2.6 (2.7–10.6)	4.7 ± 2.7 (2.4–10.6)
	Sham	3.9 ± 1.1 (2.7–6.2)	3.8 ± 0.7 (2.7–4.8)
TD			
	Cerebellar	10.5 ± 9.3 (4.9–33.3)	8.4 ± 5.7 (3-19.2)
	Sham	7 ± 2.6 (3.7–11.1)	6.5 ± 2.5 (3-10.5)
TS			
	Cerebellar	9.8 ± 5.7 (4-19.8)	8.1 ± 5.6 (2.8–18.6)
	Sham	6.4 ± 2 (4.5–9.9)	7.4 ± 4.2 (3.6–16.4)
Dx			
	Cerebellar	12.1 ± 10.5 (2.7–37.7)	11.7 ± 14.8 (3-50.1)
	Sham	9.6 ± 4.2 (3.6–15.6)	8.9 ± 3.7 (3.7–14.1)
Sx			
	Cerebellar	13.7 ± 7.9 (3.6–29.6)	12.2 ± 7.7 (2.7–25)
	Sham	9.9 ± 3.9 (3.4–14.5)	9.8 ± 3.9 (3.6–14.6)
LPDx			
	Cerebellar	0.2 ± 0.1 (0-0.4)	0.3 ± 0.1 (0.2-0.4)
	Sham	0.3 ± 0.1 (0.2-0.5)	0.4 ± 0.1 (0.2-0.5)
LPSx			
	Cerebellar	0.3 ± 0.1 (0.1-0.4)	0.4 ± 0.1 (0.2-0.5)
	Sham	0.4 ± 0.1 (0.2-0.6)	0.4 ± 0.1 (0.3-0.6)
AmP			
	Cerebellar	0.2 ± 0.1 (0.1-0.2)	0.2 ± 0.1 (0.1-0.2)
	Sham	0.1 ± 0 (0.1-0.2)	0.1 ± 0 (0.1-0.2)
DPDx			
	Cerebellar	188.6 ± 342.9 (49.7–1102)	78.5 ± 18.6 (51.6–103)
	Sham	72.5 ± 19.5 (44.9–107)	71.8 ± 19.3 (43.6–106)
DPSx			
	Cerebellar	77.4 ± 17.1 (51.7–103)	78.6 ± 17.9 (52.7–103)
	Sham	73 ± 20 (44.7–108)	72.1 ± 19.4 (44–106)
LPDxO			
	Cerebellar	0.3 ± 0.1 (0-0.4)	0.4 ± 0.1 (0.2-0.4)
	Sham	0.4 ± 0.1 (0.3-0.5)	0.4 ± 0.1 (0.3-0.5)
LPSxO			
	Cerebellar	0.3 ± 0.1 (0.1-0.4)	0.4 ± 0.1 (0.3-0.4)
	Sham	0.4 ± 0.1 (0.2-0.5)	0.4 ± 0.1 (0.3-0.4)
AmPO			
	Cerebellar	0.2 ± 0.1 (0.1-0.2)	0.2 ± 0 (0.1-0.2)
	Sham	0.1 ± 0 (0.1-0.2)	0.1 ± 0 (0.1-0.2)
DPODx			
	Cerebellar	76.9 ± 18.2 (49.8–101)	79.9 ± 19.3 (51.2–103)
	Sham	73 ± 20.3 (45.1–109)	72.5 ± 20.7 (44–106)
DPOSx			
	Cerebellar	76.6 ± 17.5 (50.8–99)	80.5 ± 17.8 (52.2–103)
	Sham	72.9 ± 19.9 (44.9–107)	72.7 ± 20.8 (44–106)

*Notes* AmP = Step Amplitude; AmPO = Step Amplitude-Obstacles; AP = Anterior-Posterior; DPDx = Right Step Distribution; DPODx = Right Step Distribution-Obstacles; DPOSx = Left Step Distribution-Obstacles; DPSx = Left Step Distribution; Dx = Right Leg; LPDx = Right Step Length; LPDxO = Right Step Length-Obstacles; LPSx = Left Step Length; LPSxO = Left Step Length-Obstacles; ML = Medio-Lateral; OAD = Eyes Open Right; OAS = Eyes Open Left; OCD = Eyes Closed Right; OCS = Eyes Closed Left; SupCoP = Postural Control Surface; Sx = Left Leg; TD = Tandem Right; TS = Tandem Left

**Fig. 2:**
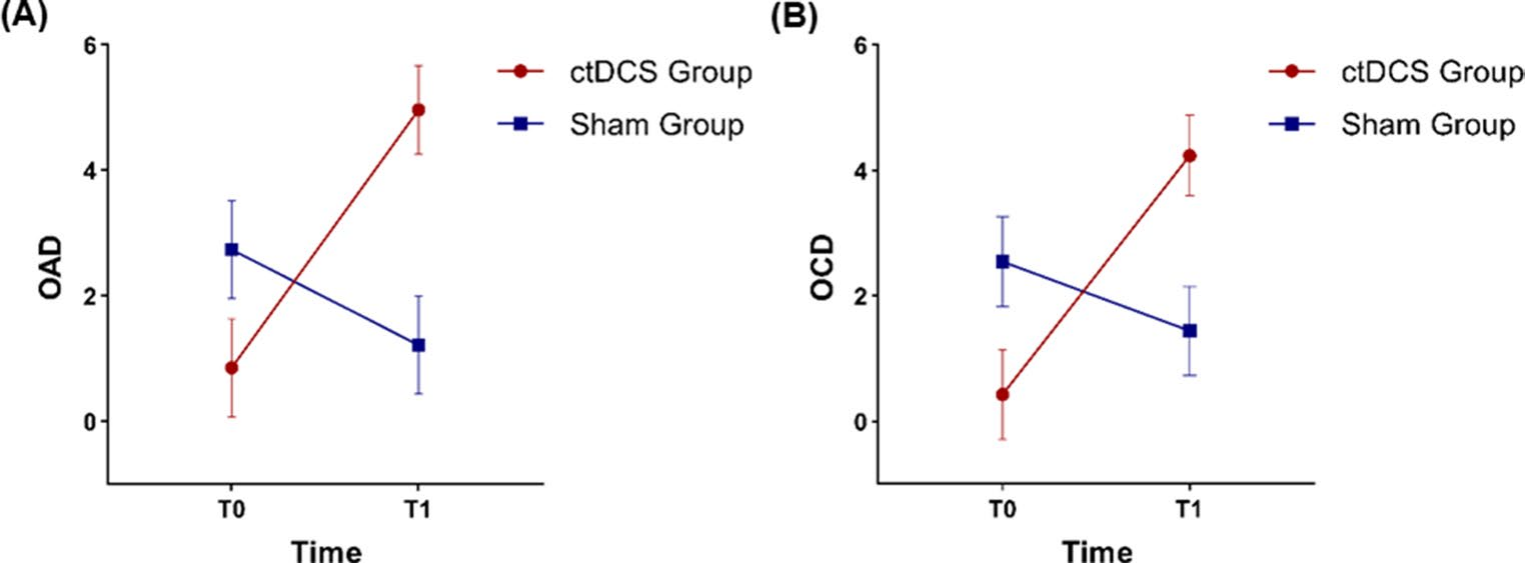
Effects plot displaying the mean scores and standard error bars related to right lower limb sway in static conditions (**A**) with eyes open (OAD) and (**B**) with eyes closed (OCD) as detected by the C-Mill

As to OAD values, a significant effect of a significant Time*Group interaction was found, with post-hoc comparisons revealing two significant differences: the first was solely and selective driven by a significant increase (*p* = .007) from T0 (*M* = 0.85; *SE* = 0.78) to T1 (*M* = 4.96; *SE* = 0.70) in the Cerebellar group. The second was solely and selective driven by group (*p* = .008) from the Cerebellar group (*M* = 4.96; *SE* = 0.70) when compared with the Sham group (*M* = 1.21; *SE* = 0.78) (Fig. 2).

As to the OCD, whilst a significant Time*Group interaction was detected as well, the *a posteriori* decomposition of this term also revealed two significant differences: the first was solely and selective driven by a significant increase (*p* = .005) from T0 (*M* = 0.43; *SE* = 0.71) to T1 (*M* = 4.24; *SE* = 0.64) in the Cerebellar group. The second was solely and selective driven by group (*p* = .041) from the Cerebellar group (*M* = 4.24; *SE* = 0.64) when compared with the Sham group (*M* = 1.44; *SE* = 0.71) (Fig. 2).

As to DPSx, whilst a significant Time*Group interaction was detected as well, the *a posteriori* decomposition of this term did not reveal any significant comparison.

**Fig. 3:**
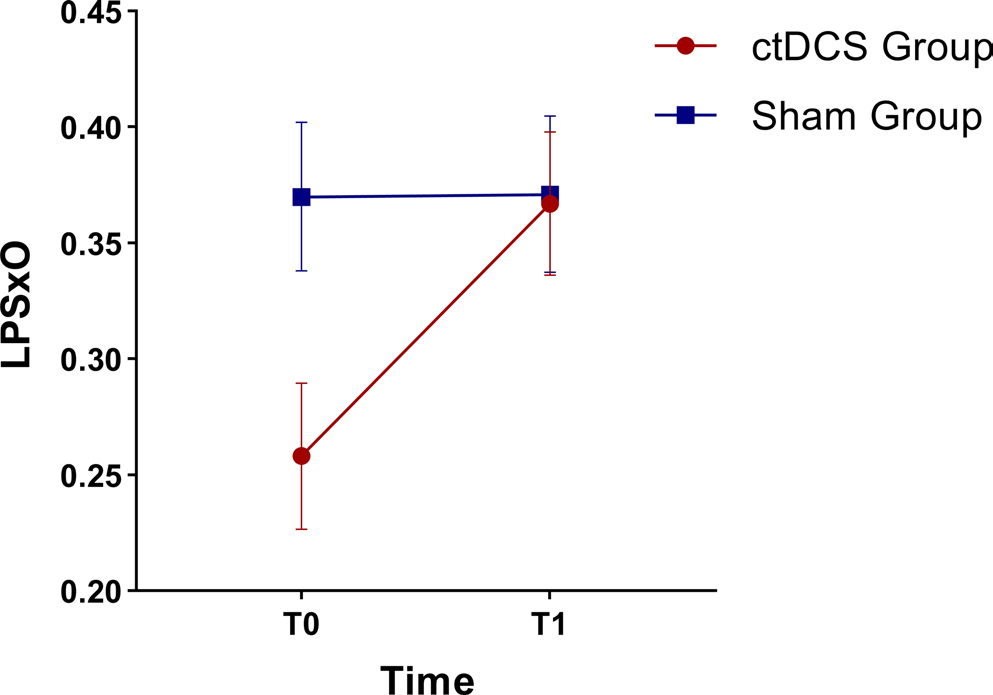
Effects plot displaying the mean scores and standard error bars for left step length under obstacle avoidance conditions (LPSxO) as detected by the C-Mill

As to LPSxO, a significant Time*Group interaction was found, with post-hoc comparisons revealing that such a significance was solely and selectively driven by a significant increase (*p* = .029) from T0 (*M* = 0.26; *SE* = 0.03) to T1 (*M* = 0.37; *SE* = 0.03) in the Cerebellar group (Fig. 3).

As to the DPODx, whilst a significant Time*Group interaction was detected as well, the *a posteriori* decomposition of this term did not reveal any significant comparison.

In contrast, no significant interaction effects were found for the remaining variables.

## Discussion

This study arises from the need to find an effective rehabilitation approach for the treatment of FoG in individuals with PD. FoG is the primary cause of disabling falls in PD [38, 39], and despite advances in understanding this phenomenon, finding effective treatments, both pharmacologically and rehabilitatively, remains challenging [4, 16]. Among nonpharmacological therapies, aerobic exercise during treadmill training and noninvasive brain stimulation, such as tDCS, showed lasting effects on FoG [40].

In terms of gait improvement, the combination of different approaches seems to be more successful in reducing FoG than conventional training alone [4, 16]. In light of this evidence, we defined a specific rehabilitation protocol consisting of gait training on the C-Mill with augmented virtual reality combined with tDCS as a possible strategy for FoG management in the PD population.

Our results did not reveal any significant interaction between the type of stimulation and the three timepoints across the motor, functional, and cognitive variables studied. The absence of significant differences in the standardized scales between groups from baseline suggests that, in our sample, the combination of ctDCS and C-Mill training did not prove to be more effective than the C-Mill training alone for the considered outcomes. Despite this, an improving trend, although not significant, is observable, especially in the general motor function and the FoG questionnaire. The restricted sample size, while adequate for preliminary analysis, may have limited the statistical power to detect significant interactions.

Regarding C-Mill measures, and specifically to postural stability, a significant interaction was shown in the measurement of body sway in static conditions with eyes open (OAD) and with eyes closed (OCD) on the right lower limb only. In-depth analysis revealed a positive effect of combined treatment in the Cerebellar group over time, whereas sham-tDCS combined with C-Mill training failed to induce significant changes. This finding could indicate that the combination of ctDCS and C-Mill VR^+^ improved postural stability both in open-eye and closed-eye conditions. Individuals with PD typically present with asymmetric motor symptoms, including asymmetric motor function of the lower limbs [41]. This asymmetry can affect postural stability and control differently between the two lower limbs and is believed to contribute to FoG pathogenesis [42]. This could account for the reduction observed in body sway during static conditions, where improvements were noted exclusively for the right lower limb. Furthermore, the improvement observed with eyes closed indicates enhanced proprioception, which is crucial for maintaining balance without visual input [43].

Additionally, healthy individuals adjust their gait speed proportionally to environmental constraints, whereas people with FoG exhibit an exaggerated response to visual information, resulting in significant reductions in gait speed and step length [39, 44, 45]. The cerebellum plays a crucial role in integrating sensory information and coordinating motor output. By enhancing cerebellar function through tDCS, it becomes possible to improve the ability to process environmental constraints accurately, thereby reducing maladaptive responses that contribute to FoG. The improvements in postural stability observed in the Cerebellar group may indicate that ctDCS combined with the C-Mill VR^+^ can modulate the exaggerated visual response in individuals with FoG. The enhanced static postural control with both eyes open and closed suggested that the combined treatment improved overall sensory integration and balance mechanisms, potentially reducing the occurrence and the severity of FoG episodes. These improvements in C-Mill outcomes may be attributed to enhanced neuroplasticity and cerebellar modulation facilitated by ctDCS, which likely improved sensory integration and motor learning. The enhanced cerebellar function may also contribute to better postural stability, enabling individuals with FoG to maintain balance in both static and dynamic conditions. This could reduce the risk of falls, enhance functional mobility, and enable individuals to navigate their environment more safely and confidently. As a result, performance in activities of daily living, such as walking, standing, and transitioning between positions, may improve, ultimately enhancing the overall quality of life for individuals with PD and FoG. Further research is warranted to explore patient-reported outcomes and quality of life measures to gain deeper insight into how these improvements impact daily living.

An explanation for the lack of significant findings with standardized scales, despite the improvements observed in C-Mill VR^+^ outcomes, could lie in the ability of the C-Mill to detect fine parameters that are not appreciated by standardized scales for several reasons. Motor and gait parameters are recorded in real time as a person walks, reacts to obstacles, and performs dual tasks. This allows immediate changes in motor control and adaptation to be observed. Standardized scales used to assess motor, functional and cognitive symptoms in individuals with PD often rely on clinical observations and self-assessments, which may not capture subtle variations or changes that occur in real time during physical activity. Additionally, the variability in disease progression and symptom severity among participants could have further blurred potential effects, masking the benefits of the combined intervention. Stratifying participants by disease severity or progression could help uncover differential responses to the intervention; also, personalized treatment protocols tailored to individual profiles may further enhance treatment effectiveness.

This study has several limitations, including the small sample size, which limits the statistical power to detect significant interactions and the generalizability of the results. However, the sample size was determined based on prior studies with comparable experimental designs [46, 47] found in the literature, which also utilized samples with a similar number of subjects. Another limitation pertains to the existing variability of stimulation protocols in the literature [22, 23], which results in uncertainty about the optimal parameters related to dose, intensity, and montage for ctDCS. Additionally, the duration of the intervention protocol may not have been sufficient to induce the expected effects. Future research should aim to optimize the ctDCS protocol for clinical practice and enhance treatment efficacy. For instance, extending the duration of treatment could help determine longer-term benefits of this combined treatment.

## Conclusions

This study aimed to test the effectiveness of a novel rehabilitative program for FoG. The program combines cerebellum-targeted tDCS with augmented reality treadmill exercises that integrates dual-tasks simulating real-world scenarios and activities of daily living. Although the clinical variables did not significantly change, the C-Mill outcomes seem to suggest that the combined treatment may enhance motor control. Notably, participants who received ctDCS and augmented reality motor-cognitive training exhibited better postural stability. The potential effectiveness of this combined rehabilitation approach should be further explored in larger studies with extended rehabilitation and cerebellar stimulation protocols.

## Electronic supplementary material

Below is the link to the electronic supplementary material.

Supplementary Material 1

## Data Availability

The datasets used and analyzed during the current study are available from the corresponding author upon reasonable request.
