# Supplementary material for: Cerebellar tDCS combined with augmented reality treadmill for freezing of gait in Parkinson’s disease: a randomized controlled trial
Source: J Neuroeng Rehabil. 2024 Sep 28;21:173. doi: 10.1186/s12984-024-01457-z (PMC11438075; doi:10.1186/s12984-024-01457-z)
Supplement: Supplementary file 1 — Supplementary Material 1 [file 12984_2024_1457_MOESM1_ESM.docx]

**Supplemental Table 1.** Results of the models testing the effects of *Time*, *Group* and their interaction on psychological and motor-functional outcomes.

| **Outcome** | **Term** | ***F/X^2^*** | ***p*** |
| --- | --- | --- | --- |
|  |  |  |  |
| **UPDRS-III** |  |  |  |
|  | *Time* | **19.32** | <.001 |
|  | *Group* | 2.60 | .128 |
|  | *Time*Group* | .84 | .444 |
| **FoG-Q** |  |  |  |
|  | *Time* | **17.24** | <.001 |
|  | *Group* | .18 | .676 |
|  | *Time*Group* | .92 | .410 |
| **MoCA** |  |  |  |
|  | *Time* | .19 | .829 |
|  | *Group* | .04 | .843 |
|  | *Time*Group* | .03 | .974 |
| **FAB** |  |  |  |
|  | *Time* | 2.80 | .077 |
|  | *Group* | .02 | .883 |
|  | *Time*Group* | .72 | .497 |
| **PDQ-8** |  |  |  |
|  | *Time* | .22 | .806 |
|  | *Group* | .50 | .490 |
|  | *Time*Group* | .24 | .790 |
| **BDI** |  |  |  |
|  | *Time* | .60 | .557 |
|  | *Group* | .94 | .348 |
|  | *Time*Group* | .29 | .749 |
| **BORG_post** |  |  |  |
|  | *Time* | .24 | .790 |
|  | *Group* | 2.26 | .154 |
|  | *Time*Group* | 1.87 | .172 |
| **Mini-BEST** |  |  |  |
|  | *Time* | **14.68** | <.001 |
|  | *Group* | 1.40 | .255 |
|  | *Time*Group* | .87 | .430 |
| **6MWT** |  |  |  |
|  | *Time* | **4.21** | .024 |
|  | *Group* | **7.13** | .017 |
|  | *Time*Group* | 1.08 | .352 |
| **IADL** |  |  |  |
|  | *Time* | .88 | .424 |
|  | *Group* | .97 | .339 |
|  | *Time*Group* | .88 | .424 |
| **BI** |  |  |  |
|  | *Time* | **19.20** | <.001 |
|  | *Group* | **15.15** | <.001 |
|  | *Time*Group* | 5.13 | .077 |
| **TUG** |  |  |  |
|  | *Time* | 2.88 | .237 |
|  | *Group* | 3.42 | .065 |
|  | *Time*Group* | 2.49 | .288 |
| **ADL** |  |  |  |
|  | *Time* | 1.35 | .509 |
|  | *Group* | **16.83** | <.001 |
|  | *Time*Group* | .82 | .663 |
| **MMSE** |  |  |  |
|  | *Time* | .03 | .983 |
|  | *Group* | **.**00 | .964 |
|  | *Time*Group* | .02 | .988 |

**Notes.** ADL=Activity of Daily Living; BDI=Beck Depression Inventory; BI=Barthel index; FAB=Frontal Assessment Battery; FoG-Q=Freezing of Gait Questionnaire; IADL=Instrumental Activity of Daily Living; MoCA=Montreal Cognitive Assessment; MMSE=Mini Mental State Examination; Mini-BEST=Mini Balance Evaluation System Test; 6MWT=6 Minute Walking Test; PDQ-8=Parkinson’s Disease Questionnaire; TUG=Time Up and Go Test; UPDRS-III=Unified Parkinson’s Disease Rating Scale–Part III.

**Supplementary Material 2.** Results of the models testing the effects of *Time*, *Group* and their interaction on C-Mill outcomes.

| **Outcome** | **Term** | ***F/X^2^*** | ***p*** |
| --- | --- | --- | --- |
|  |  |  |  |
| **SupCoP** |  |  |  |
|  | *Time* | .84 | .368 |
|  | *Group* | .44 | .511 |
|  | *Time*Group* | .02 | .889 |
| **AP** |  |  |  |
|  | *Time* | .01 | .910 |
|  | *Group* | .38 | .549 |
|  | *Time*Group* | .62 | .441 |
| **OAD** |  |  |  |
|  | *Time* | 2.90 | .099 |
|  | *Group* | 1.51 | .229 |
|  | *Time*Group* | **13.71** | <.001 |
| **OCD** |  |  |  |
|  | *Time* | 4.01 | .062 |
|  | *Group* | .23 | .639 |
|  | *Time*Group* | **13.18** | .002 |
| **Sx** |  |  |  |
|  | *Time* | .73 | .406 |
|  | *Group* | 1.46 | .246 |
|  | *Time*Group* | .67 | .425 |
| **LPDx** |  |  |  |
|  | *Time* | 4.46 | .051 |
|  | *Group* | 2.36 | .146 |
|  | *Time*Group* | 1.10 | .310 |
| **LPSx** |  |  |  |
|  | *Time* | **8.51** | .010 |
|  | *Group* | 1.27 | .278 |
|  | *Time*Group* | 2 | .177 |
| **AmP** |  |  |  |
|  | *Time* | **6.15** | .025 |
|  | *Group* | 4 | .064 |
|  | *Time*Group* | .04 | .839 |
| **DPSx** |  |  |  |
|  | *Time* | 0 | .973 |
|  | *Group* | .37 | .555 |
|  | *Time*Group* | **10.38** | .006 |
| **LPDxO** |  |  |  |
|  | *Time* | 2.72 | .121 |
|  | *Group* | 2.30 | .151 |
|  | *Time*Group* | 3.14 | .098 |
| **LPSxO** |  |  |  |
|  | *Time* | **5.61** | .034 |
|  | *Group* | 2.26 | .156 |
|  | *Time*Group* | **5.40** | .037 |
| **AmPO** |  |  |  |
|  | *Time* | .17 | .684 |
|  | *Group* | 4.56 | .050 |
|  | *Time*Group* | 3.78 | .073 |
| **DPODx** |  |  |  |
|  | *Time* | .25 | .624 |
|  | *Group* | **.**28 | .624 |
|  | *Time*Group* | **8.11** | .014 |
| **DPOSx** |  |  |  |
|  | *Time* | .58 | .460 |
|  | *Group* | .31 | .584 |
|  | *Time*Group* | 10.46 | .007 |
| **OAS** |  |  |  |
|  | *Time* | .84 | .360 |
|  | *Group* | .32 | .574 |
|  | *Time*Group* | .96 | .328 |
| **OCS** |  |  |  |
|  | *Time* | .12 | .733^b^ |
|  | *Group* | .45 | .504^b^ |
|  | *Time*Group* | .02 | .884^b^ |
| **TD** |  |  |  |
|  | *Time* | **7.44** | .006^b^ |
|  | *Group* | .46 | .499^b^ |
|  | *Time*Group* | 2.27 | .132^b^ |
| **TS** |  |  |  |
|  | *Time* | 1.12 | .289^b^ |
|  | *Group* | .47 | .493^b^ |
|  | *Time*Group* | 3.30 | .069^b^ |
| **Dx** |  |  |  |
|  | *Time* | **4.03** | .045^b^ |
|  | *Group* | 0 | .993^b^ |
|  | *Time*Group* | 1.10 | .294^b^ |
| **DPDx** |  |  |  |
|  | *Time* | 1.63 | .202 |
|  | *Group* | .66 | .416 |
|  | *Time*Group* | 1.38 | .240 |
| **MLr** |  |  |  |
|  | *Time* | .09 | .761 |
|  | *Group* | 1.77 | .183 |
|  | *Time*Group* | .15 | .701 |

**Notes.** AmP=Step Amplitude; AmPO=Step Amplitude-Obstacles; AP=Anterior-Posterior; DPDx=Right Step Distribution; DPODx=Right Step Distribution-Obstacles; DPOSx=Left Step Distribution-Obstacles; DPSx=Left Step Distribution; Dx=Right Leg; LPDx=Right Step Length; LPDxO=Right Step Length-Obstacles; LPSx=Left Step Length; LPSxO=Left Step Length-Obstacles; ML=Medio-Lateral; OAD=Eyes Open Right; OAS=Eyes Open Left; OCD=Eyes Closed Right; OCS=Eyes Closed Left; SupCoP=Postural Control Surface; Sx=Left Leg; TD=Tandem Right; TS=Tandem Left.
